# The Microtubule-Associated Protein Tau and Its Relevance for Pancreatic Beta Cells

**DOI:** 10.1155/2016/1964634

**Published:** 2015-12-28

**Authors:** Magdalena Maj, Gregor Hoermann, Sazan Rasul, Wolfgang Base, Ludwig Wagner, Johannes Attems

**Affiliations:** ^1^Department of Internal Medicine III, Division of Nephrology and Dialysis, Medical University of Vienna, 1090 Vienna, Austria; ^2^Department of Laboratory Medicine, Medical University of Vienna, 1090 Vienna, Austria; ^3^Institute of Neuroscience, Newcastle University, Newcastle upon Tyne NE4 5PL, UK

## Abstract

Structural and biochemical alterations of the microtubule-associated protein tau (MAPT) are associated with degenerative disorders referred to as tauopathies. We have previously shown that MAPT is present in human islets of Langerhans, human insulinomas, and pancreatic beta-cell line models, with biophysical similarities to the pathological MAPT in the brain. Here, we further studied MAPT in pancreatic endocrine tissue to better understand the mechanisms that lead to functional dysregulation of pancreatic beta cells. We found upregulation of MAPT protein expression in human insulinomas when compared to human pancreatic islets of Langerhans and an imbalance between MAPT isoforms in insulinomas tissue. We cloned one 3-repeat domain MAPT and transduced this into a beta-cell derived rodent cell line Rin-5F. Proliferation experiments showed higher growth rates and metabolic activities of cells overexpressing MAPT protein. We observed that a MAPT overexpressing cell line demonstrates altered insulin transcription, translation, and insulin secretion rates. We found the relative insulin secretion rates were significantly decreased in a MAPT overexpressing cell line and these findings could be confirmed using partial MAPT knock-down cell lines. Our findings support that MAPT may play an important role in insulin granule trafficking and indicate the importance of balanced MAPT phosphorylation and dephosphorylation for adequate insulin release.

## 1. Introduction

Pancreatic neuroendocrine cells are located within the islets of Langerhans and are involved in glucose metabolism in mammals thereby regulating blood glucose levels. On the one hand, loss of pancreatic beta cells, which is observed in diabetes mellitus type 1 and in advanced stages of diabetes mellitus type 2, leads to decreased insulin production. On the other hand, uncontrolled proliferation of pancreatic neuroendocrine cells in pancreatic neuroendocrine tumors (PanNETs) results in inadequate hormone secretion [1]. PanNETs are rare, accounting for less than 5% of pancreatic neoplasias yet they represent an important clinical form of pancreatitis [2]. The most common PanNETs are insulinomas with a yearly incidence of 1–4 per 1.000.000 [3]. However, it was suggested that insulinomas often remain undiagnosed during life, as their prevalence in autopsy studies reaches up to 10%. Insulinomas arise in the islets of Langerhans and usually measure less than 2 cm in diameter. Their increased insulin production frequently results in hypoglycemia.

Although diabetes and PanNETs represent an opposite end of the spectrum with respect to blood glucose levels, the level of secreted insulin is crucial in both conditions. Therefore, the elucidation of mechanisms and molecules, which play a role in the control of insulin synthesis and secretion, is paramount in order to further our understanding of the pathophysiology of both diabetes and PanNETs.

In the present study, we focus on the microtubule-associated protein tau (MAPT). Together with MAP2 and MAP4 [4, 5], MAPT belongs to the family of microtubule-associated proteins, which stabilize microtubule tracks [6–8]. Microtubules represent the rails for neurotransmitter vesicle trafficking towards the synapses [9] and for insulin granules maturation, processing, and prepriming them for exocytosis [10–14]. MAPT is present not only in the central nervous system (CNS) but also in pancreas, breast, prostate, and renal tubules [15, 16] (http://www.proteinatlas.org/).

Aggregates of abnormally hyperphosphorylated MAPT are the main constituent of neurofibrillary tangles (NFTs) and neuropil threads (NTs) that are characteristic neuropathological hallmark lesions of Alzheimer's disease (AD) [17, 18]. Interestingly, it has been recently reported that abnormally hyperphosphorylated MAPT was found in pancreatic islets of individuals suffering from diabetes mellitus type 2 [19]. In our previous study, we described the biochemistry of MAPT in the beta-cell derived rodent cell line Rin-5F [20].

Motivated by these findings, one part of the present study aimed to evaluate MAPT expression levels in human pancreatic islets and human insulinomas. In the second part, we investigated the relevance of MAPT protein for beta cells using an* in vitro* pancreatic cell line model.

The process of insulin secretion has been studied in isolated rodent islets [21–23] and beta-cell derived insulinoma cell lines [24, 25]. Although the coupling between stimulus and secretion is disrupted in most cell lines [26, 27], the mode of granule trafficking from immature to preprimed and primed granules prepared for secretion might be preserved. The granule maturation process as described for beta cells [28, 29] and intracellular movement of insulin/transmitter containing vesicles depends on a functioning microtubular machinery together with motor proteins and microtubule-associated proteins such as the MAPT [30].

Overexpression of human MAPT isoforms within neurons has been studied using experimental animal models [31–33]. These studies have demonstrated that MAPT overexpression leads to axonopathy and has an influence on MAPT compartmentalization as well as hyperphosphorylation.

Therefore, in the present study, we aimed to investigate the effects of MAPT overexpression on pancreatic beta-cell line growth rates and on insulin transcription, translation, and insulin secretion rates. We also looked at the effects of knocking down the endogenous MAPT. Our data indicate the importance of MAPT levels and MAPT phosphorylation status for pancreatic beta cells' biology.

## 2. Material and Methods

### 2.1. RNA Isolation

#### 2.1.1. Human Islets and Insulinomas Tissue

Total RNA was isolated from human insulinoma tissues stored frozen after surgery in liquid nitrogen. Briefly, about 50–100 mg of tumor tissue was homogenized in 1 mL TRIzol (Invitrogen, Lofer, Austria) and total RNA was isolated by phase-separation and precipitation with isopropanol following the manufacturers' instructions. Human pancreatic islet total RNA was obtained from the Department of Medical Biochemistry and Genetics, University of Copenhagen (Denmark) (Professor J. H. Nielsen). One *μ*g of total RNA was reverse transcribed using SuperScript II (Invitrogen) and random primers and the resultant cDNA was diluted 1 : 3 in H_2_O. Of note, this part of the study had been approved by the Ethics Committee, Medical University of Vienna.

#### 2.1.2. Pancreatic Beta-Cell Derived Rodent Cell Line Rin-5F

We choose the rat insulinoma cell line, Rin-5F, a clone derived from the Rin-m rat islet line (ATTC CRL-2057), as our main investigative tool for* in vitro* analysis. This cell line represents a model to study the biology of pancreatic beta cells, specifically the mechanism controlling the synthesis, storage, and secretion of insulin.

Rin-5F cell lines were cultured in complete medium [20] and harvested into 900 *μ*L TRIzol. The RNA and cDNA were processed and obtained in the same way as described above.

### 2.2. RT-qPCR

#### 2.2.1. Human Islets and Insulinomas

In order to measure all MAPT isoforms in human pancreatic islets and insulinomas, we designed* Pan MAPT* specific primers:* MAPT* fw: (5′-CCTCTCCCGTCCTCGCCTCTG-3′) and* MAPT* rv: (5′-GGGTCAGCCATCCTGGTTCA-3′). One *μ*L of islet or insulinoma cDNA and five *μ*L of the SYBR Green/RQX PCR Master Mix (SA Bioscience, Cat. number PA-012), together with 10 *μ*M* Pan MAPT* primers, were used to measure the relative MAPT gene expression levels. The RT-qPCR was run on the StepOnePlus PCR machine and calculated using the ΔΔCT method. Actin was used as a house-keeping gene.

#### 2.2.2. Pancreatic Beta-Cell Derived Rodent Cell Line Rin-5F

To evaluate individual gene expression levels TaqMan specific probes from Applied Biosystems (the human* MAPT*, number: Hs00213487_m1 MAPT, Lot.: 471108; the rat* MAPT*, number: Rn01495715_m1, Lot.: 612900; the Rat* Insulin*, number: Rn02121433_g1 Ins1, Lot.: 771584), together with TaqMan 2xPCR Master Mix (Applied Biosystems, Lot.: N10545), were used for RT-qPCR measurements on the StepOnePlus PCR machine. After normalization of individual samples using* Gapdh* (number: Rn01775763_g1 Gapdh, Lot.: 740946) and/or* Pdx1* (number: Rn00755591_m1 Pdx1, Lot.: 455683) as house-keeping genes, the expression levels were calculated with the ΔΔCT method taking the sham-transfectant or wild type line as standard.

### 2.3. PCR for Differentiation 3RD versus 4RD Tau Isoforms

For differentiating 3-repeat domain (3RD) versus 4-repeat domain (4RD) MAPT isoforms in human pancreatic islets and insulinomas, we used the following primers: HE9F2 (5′-ACCCAAGTCGCCGTCTTCCGCC-3′) and HE11R (5′-CACCTTGCTCAGGTCAACTGGT-3′) specific for exons 9 and 11, respectively [34]. This revealed PCR products of 170 bp when excluding exon 10 (E10−, which corresponds to 3RD MAPT isoforms) and 256 bp when including exon 10 (E10+, which corresponds to 4RD MAPT isoforms). The PCR conditions were 35 cycles: 95°C, 68°C, and 72°C with 7 min extension at the end, using high fidelity polymerase (Roche, Basel, Switzerland). In order to measure the product within the linear amplification phase, the PCR reaction was terminated between cycles 27 and 29. Product separation was carried out at a 1.5% agarose gel and band intensity was quantitated using Lumi Imager F1 (Roche) and Image J program.

### 2.4. Cloning of Human Tau Isoforms

Human cDNA (as template) together with the forward primer 5′-ATGGCTGAGCCCCGCCAG-3′ and reverse primer 5′-TCACAAACCCTGCTTGGCCA-3′ was used for amplification of the coding region of human* MAPT*. PCR conditions were 35 cycles: 95°C for denaturing, 62.5°C for annealing, and 72°C for synthesis with 7 min extension at the end, using high fidelity polymerase (Roche). The resultant PCR product was analysed at a 1% agarose gel, the MAPT specific bands were cut out, and the DNA amplicons were bound to glass beads (at RT, o/n under constant rotation) (S&S Elu-Quik, Schleicher & Schuell, Germany). After washing, glass beads were vacuum dried and DNA was eluted into 50 *μ*L H_2_O (2 min at 50°C).

The DNA fragments (3 *μ*L) were ligated into the pGEM-T Easy vector system following manufacturer's instructions (Promega, Madison, WI, USA). The ligation product (2 *μ*L) was transformed into One Shot TOP10 Chemically Competent* E. coli* (Invitrogen) with heat-shock method following manufacturer's instruction and grown on LB/Amp/X-gal plates (o/n at 37°C). White colonies were inoculated into TY/Amp medium, grown for 16 h at 37°C, and plasmids were isolated by the miniprep method (Qiagen) and the inserts were identified by restriction enzymes digest (*EcoR I*) and sequencing.

For engineering of the protein expression vector, a novel forward primer including CACC at the 5′ end was designed, and the 3RD-MAPT3 isoform encoding pGEM-T Easy plasmid was chosen as template. The resultant PCR product was cloned into the pENTR/D-TOPO vector as indicated in the manufacturer's test manual (Life Technologies). The recombinant vector was transformed into One Shot TOP10 Chemically Competent* E. coli* (Invitrogen) and grown o/n on LB/Kanamycin plates. Outgrowing colonies were inoculated in TY/Kanamycin medium, grown for 16 h; plasmids were isolated by the miniprep method (Qiagen) and inserts were identified by restriction enzymes using* Hind III* and* Not I*. The resultant entry clone was used for transferring the insert into the expression/destination vector: pEXP1-DEST (Invitrogen). The LR clonase reaction was carried out at 25°C as indicated in the test manual (in brief: 2 *μ*L LR clonase II was mixed with 150 ng Tau pENTR/D-TOPO plasmid and 150 ng wild type pEXP1-DEST vector in a total volume of 10 *μ*L) and transformed into chemically competent cells using the heat-shock method as indicated above. Outgrowing colonies were inoculated in TY medium, grown for 16 h at 37°C; insert-containing plasmid was verified (by restriction enzyme digest* Hind III*), transformed into BL21 codon plus bacteria, and cloned on TY/Amp plates. A single colony was inoculated into an Erlenmeyer flask in 25 mL TY/Amp medium o/n. For induction of protein expression, the IPTG (100 *μ*M) was added (6 hr incubation at 31°C under constant rotation). The bacterial culture was harvested by centrifugation at 3200 rpm for 12 min and the resultant pellet was lysed in 4 M Urea, 100 mM phosphate, and pH 8 lysis buffer. The cell debris was separated by centrifugation at 10000 rpm on a J2-MC centrifuge (Beckman) at 4°C and the obtained supernatant was incubated (1 hr at 4°C under constant rotation) with the Ni-NTA His-Bind Resin preequilibrated in lysis buffer. Loaded beads were washed three times using 1x Ni-NTA washing buffer (300 mM NaCl, 50 mM sodium phosphate buffer, 20 mM imidazole, and pH 8.0). MAPT protein was eluted using 1x Ni-NTA elution buffer (300 mM NaCl, 50 mM sodium phosphate buffer, 250 mM imidazole, and pH 8.0). Eluted fractions were tested for protein content and size by SDS-PAGE and Coomassie Blue staining.

For generating a mammalian expression plasmid, the clonase reaction was performed using pENTR/D-TOPO MAPT encoding plasmid together with pMSCV empty vector similarly as described above. Briefly, 150 ng pENTR/D-TOPO MAPT was combined with 150 ng pMSCV empty vector in 6 *μ*L TE buffer including 2 *μ*L clonase II in a final volume of 10 *μ*L. The clonase reaction was transformed into One Shot TOP10 chemically competent* E. coli* (Invitrogen). Clones were selected on TY/Amp plates and grown out in TY medium (16 h at 37°C) and the pMSCV Tau plasmid was purified using Qiagen columns.

### 2.5. Generation of MAPT Encoding Retroviral Particles

Recombinant retroviruses were produced by transient transfection using HEK-293FT cells (Invitrogen). HEK-293FT cells were transfected with retroviral vector pMSCV MAPT (3RD-MAPT3) and plasmids encoding GAG-POL and VSV-G using lipofectamine 2000 (Invitrogen), according to the recommendation of the manufacturer.

The Rin-5F cells were used for the retroviral-mediated overexpression of MAPT. Semiconfluent Rin-5F cells were retrovirally transduced by spin infection (800 ×g, 90 min, 32°C) in the presence of polybrene (7 *μ*g/mL). Four days after transduction, cells were treated with puromycin (1 *μ*g/mL) for chemoselection. The culture supernatant was exchanged every third day and outgrowing cells were passaged every fourth day.

Sham-transfectant Rin-5F cells were obtained by transducing Rin-5F cells using empty pMSCV vector and chemoselecting for resistant cells with puromycin (1 *μ*g/mL). This cell line was used for comparative reason and is named later an* Empty* cell line.

### 2.6. Tau Knockdown

For RNA interference experiments, the siRNA encoding lentivirus compatible plasmids were purchased from Thermo Scientific (Cat. number: RHS4533, Clone numbers: TRCN0000083973, TRCN0000083974, TRCN0000083975, and TRCN0000083976). The four plasmids were separately amplified in* E. coli* and used for generation of siRNA encoding virus particles. Recombinant lentiviruses were produced as described previously [35].

The wild type Rin-5F cell line was transduced with each of the* Mapt* siRNAs as described above. Individual cell lines were grown out following chemoselection with puromycin (1 *μ*g/mL).

### 2.7. Immunofluorescence

Cytospin preparations of MAPT overexpressing and Empty cell lines were fixed in acetone for 5 min, blocked with RPMI 1640 medium containing 10% FCS for 10 min, and subsequently stained with the polyclonal rabbit anti-human Tau Ab (Cat. number A 0024, DakoCytomation, Denmark) (diluted 1 : 200 in PBS) over night at 4°C. Following washing in 0.05% (v/v) Tween-PBS for 15 min, a secondary Ab was applied, the goat anti-rabbit Alexa 488 (Cat. number P0448, DakoCytomation) (diluted 1 : 200 in PBS), and incubated for 2 h at RT. The slides were mounted with Vectashield (Vector Laboratories, Burlingame, CA, USA). Images were taken using the Axiovert confocal microscope 200 M (Zeiss Jena, Germany) and processed using LSM 5 software (Zeiss).

### 2.8. Immunoblotting

The Western blotting was essentially performed as described in [20]. In brief, 2 × 10^6^ Rn-5F cells (Empty and MAPT overexpressing cell lines: not treated or treated for 1 h with either 1 *μ*M or 1 nM Wortmannin or with dimethyl sulfoxide (DMSO) as a control) were lysed individually in 100 *μ*L Weinberg buffer (50 mM Hepes, pH 7.0, 0.5% Nonidet P-40, 250 mM NaCl, and 5 mM EDTA) containing phenylmethylsulfonyl fluoride (10 mM), sodium vanadate (10 mM), and *β*-glycerophosphate (1 M). After preclearing cell lysate by centrifugation at 13000 rpm for 10 min, the supernatant was combined with SDS sample buffer and loaded onto 10% SDS-PAGE, which was then blotted onto Immobilon-P Transfer Membrane (Millipore, Vienna, Austria) using a semidry blotting device. Blocked membranes were exposed o/n at 4°C to primary antibodies: polyclonal rabbit anti-human Tau (dilution 1 : 5000, Cat. number A 0024, DakoCytomation, Denmark, Glostrup), anti-human PHF-Tau mAb, clone AT8 (dilution 1 : 2500, Lot. number FK 93121, Pierce, Rockford, IL, USA), and mouse monoclonal anti-*β*-actin (dilution 1 : 5000, Cat. number NB600-501, Novus Biologicals, Littleton, CO, USA). Following washing in TPBS, the HRP-conjugated goat-anti rabbit (DakoCytomation, Cat. number P0048) or goat-anti mouse (DakoCytomation, Cat. number P0047) was applied and sites of antibody binding were visualized by chemiluminescence which was recorded by Lumi Imager F1 (Roche).

### 2.9. MTT Assay

Equal numbers (2 × 10^5^) of Empty and MAPT overexpressing Rin-5F cell lines were seeded in quadruplicates onto a 24-multiwell plate and incubated with 1 mL complete medium for 36 h and 60 h. After the respective incubation period, the culture medium was replaced with RPMI containing MTT (3-(4,5-dimethylthiazol-2-yl)-2,5-diphenyltetrazolium bromide) (500 *μ*g/mL). Following 60 min incubation, the medium containing MTT was removed and the resultant formazan dye was dissolved in HCl acidified (50 mM) DMSO containing SDS (10% w/v). The colour intensity was evaluated by spectrophotometric measurement (*λ* = 562 nm).

### 2.10. Cell Proliferation by Evaluation of Cell Number

Equal numbers of Empty and MAPT overexpressing cells were plated onto culture dishes, and the cell number was evaluated by a cell counter (XE-2100 Sysmex) after 60 h. The experiment was carried out three times at different time points in triplicate.

### 2.11. Insulin Secretion Assay

(a) Empty and MAPT overexpressing Rin-5F cell lines (2 × 10^5^ cells/well) were seeded into 250 *μ*L RPMI 1640 culture medium and stimulated with 20 mM KCl (final concentration). After 2 h incubation, the supernatant was harvested and centrifuged at 1000 ×g for 5 min to pellet cells. Nonstimulated cells were included as a control. For measuring intracellular insulin content, cells were lysed using 1% (v/v) Tween-PBS.

(b) Empty and MAPT overexpressing Rin-5F cell lines (4 × 10^5^ cells/well) were seeded onto 12-well plate. After 24 h culturing to obtain cell adherence, the culture supernatant was changed by either only prewarmed RPMI 1640 or prewarmed RPMI 1640 supplemented with the Wortmannin (1 *μ*M). Following an incubation period of 2 h at 37°C, an aliquot of 200 *μ*L was harvested and kept at 4°C until measurement of insulin level. Further aliquots (in quadruplicates) were harvested at 4, 6, 10, and 24 h incubation period.

Each experiment was carried out in triplicate at three different days. 

*An Insulin RIA (Radioimmunoassay)*. An Insulin RIA (Radioimmunoassay) (Cat. number RI-13K, Millipore, Billerica, MA, USA) was used to determine insulin content of individual samples. The assay is based on the radioactive ^125^I-labeled insulin and it utilizes a Rat Insulin antiserum. The assay was performed as described in the assays' manual. In brief, standards, quality controls, and samples diluted with assay buffer, 1 : 10 (samples harvested at 1, 2, 3, and 4 h incubation), 1 : 20 (at 6 h), and 1 : 50 (at 24 h), were incubated over night at 4°C with ^125^I-Insulin Tracer and Rat Insulin Ab. On the following day, the precipitating reagent was added. After an incubation period of 20 min, the supernatant was decanted and dried tubes were measured in a gamma counter for 1 min each.

## 3. Results

### 3.1. MAPT Expression in Islets and Insulinomas

MAPT is highly expressed in the central nervous system (CNS) where it plays a crucial role in the axonal transport and neurite outgrowth [8, 36, 37]. However, MAPT was shown to be present also in other tissues [15, 16, 38] (http://www.proteinatlas.org/) like the endocrine parts of the pancreas, breast, prostate, and renal tubules.

**Figure 1:**
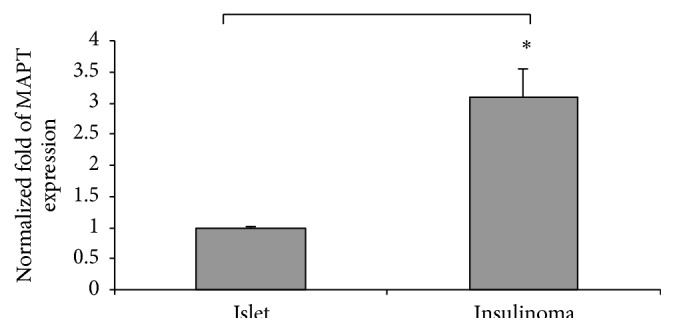
RT-qPCR of RNA isolated from human islets and human insulinomas. Expression of MAPT gene level evaluated in human islets (*n* = 3) and in human pancreatic insulinomas (*n* = 5); *p* < 0.01.

Following our previous findings that MAPT is highly expressed in pancreatic islets of Langerhans [20], we raised the question whether MAPT is expressed at the same level in the islets of Langerhans isolated from healthy organ donors versus pancreatic insulinoma tumors. For that reason, we analyzed RNA from islets of healthy organ donors (*n* = 3) and from insulinomas (*n* = 5) for reverse transcription. Obtained cDNA was used to check the* MAPT* gene expression level. Interestingly,* MAPT* expression is over 3 times higher in insulinomas when compared to islets, SD ± 0.12 (*p* < 0.01) (Figure 1).

### 3.2. MAPT Isoforms

The distortion of normal equimolar ratio between 3-repeat domain (3RD) and 4-repeat domain (4RD) MAPT isoforms is observed in human brain tauopathies. Following this, we used predesigned specific primers that recognize MAPT isoforms containing exon 10 (HE11R) and missing exon 10 (HE9F2) and subsequently performed PCR. This method has been previously shown [34] to provide different size fragments depending on whether exon 10 is out-spliced or kept within the mRNA. The fragment size is 170 bp for E10− and 265 bp for E10+. A balanced ratio = 1, SD ± 0.07 isoform expression (3RD to 4RD) was found in islets (*n* = 4), whereas an inverse ratio = 1.2, SD ± 0.18 was found in insulinoma tissue (*n* = 7), *p* < 0.05.

### 3.3. Frequency Appearance of Specific MAPT Isoforms

**Figure 2:**
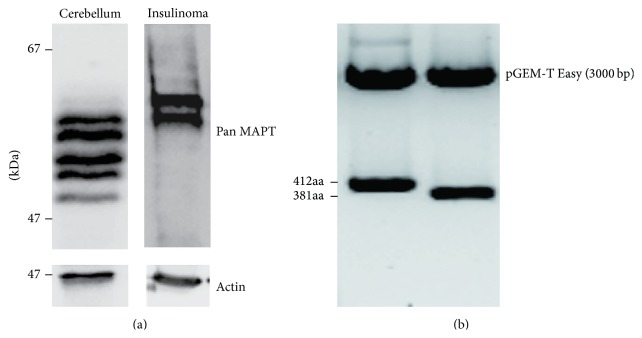
MAPT protein appearance at insulinoma tissue. (a) Comparison of MAPT protein electrophoretic pattern (10% SDS-PAGE gel) from cerebellum and human insulinoma tissue. (b) Subcloned insulinoma tumor cDNA into the pGEM-T Easy replication plasmids. Lower bands demonstrate inserts which contained exclusively the coding region of MAPT3 or MAPT4.

The six MAPT isoforms (MAPT1 to MAPT6) expression is well documented in the human brain [39, 40]. Therefore, we investigated whether the same isoforms can be found in human beta-cell tumors. For this reason, we separated insulinoma tumor and cerebellum proteins in an SDS-PAGE gel and performed pan MAPT specific immunoblotting. As shown in Figure 2(a), two distinct isoforms were the predominant isoforms in insulinoma. To further characterize these isoforms we reverse-transcribed insulinoma tumor RNA and subcloned the PCR amplified cDNA into the pGEM-T Easy replication plasmids. The inserts were sequenced and were shown to contain exclusively the coding region of MAPT3 (corresponding to MAPT3 protein: 381aa long isoform) or MAPT4 (corresponding to MAPT4 protein: 412aa long isoform) (Figure 2(b)).

### 3.4. Transgene Overexpression and Characterization of Cell Lines

The microtubular machinery (a part of which is MAPT) together with motor proteins plays a crucial role in the insulin/transmitter containing vesicles intracellular trafficking and their expulsion. To follow this, we transduced an insulinoma cell line with the 3-repeat domain MAPT, namely, MAPT3 (381aa) isoform, to test its effect on insulin secretion.

**Figure 3:**
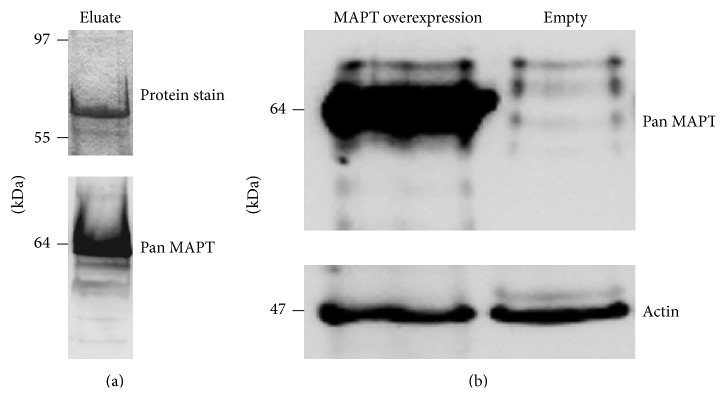
(a) Bacterial expression of MAPT isoform (3RD-MAPT3, 381aa). Ni-NTA purified human recombinant His-tagged MAPT visualized by Coomassie Blue staining (upper panel). The immunoblotting of recombinantly produced MAPT in BL21* E. coli* visualized by the pan MAPT specific Ab (lower panel). (b) Overexpression of 3RD-MAPT3 in Rin-5F cells. The immunoblotting of MAPT overexpressing Rin-5F cell line versus Empty Rin-5F cell line using a pan MAPT specific Ab. Densitometric measurements showed 10-fold stable overexpression of MAPT in the transgenic cell line.

Using human cDNA as template, we amplified MAPT encoding isoforms and cloned one of them into an* E. coli* expression plasmid. Employing* E. coli* BL21 we generated a His-tagged MAPT protein. The purified MAPT protein has been used to visualize its size of expression with our Abs. Following the successful attempt of bacterial expression (Figure 3(a)), we transferred the coding sequence of MAPT isoform 3RD-MAPT3 into a retrovirus-based mammalian expression plasmid as described in Section 2. After packaging the expression plasmid into a lentiviral particle, the Rin-5F insulinoma cell line was transduced and subsequently selected for MAPT gene overexpressing cells using puromycin. The outgrowing cell line was expanded and tested for MAPT protein overexpression (Figure 3(b)). Western blotting allowed the detection of a 10-fold stable overexpression of MAPT in the transgenic cell line when compared to the Empty cell line.

### 3.5. Immunofluorescence

**Figure 4:**
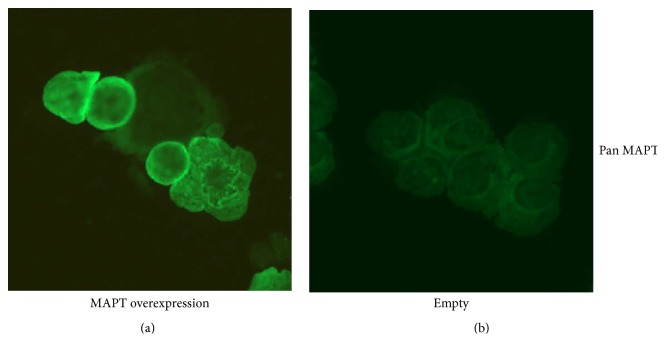
Confocal immunofluorescence staining of Rin-5F cell lines. MAPT overexpressing cell line (a) and Empty (b) stained by the pan MAPT specific Ab followed by Alexa Fluor goat anti-rabbit Ab. Images were taken by Axiovert confocal microscope 200 M (Zeiss Jena, Germany).

In order to establish the morphology and intracellular topology of the transduced versus Empty cell line confocal microscopy images were taken. Higher cytoplasmic MAPT protein staining within the MAPT overexpressing cell line but with the absence of any aggregates or tangle formation was observed. Interestingly, not 100% of the cell population showed this high level of MAPT staining, but this seemed to depend on the stage of the cell cycle (Figure 4).

### 3.6. Cell Replication

**Figure 5:**
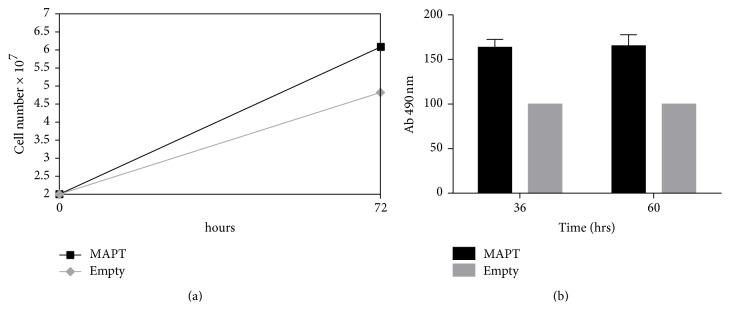
(a) Cell proliferation. Growth rate of MAPT overexpressing cell line versus Empty; *p* < 0.05. Presented data are means from three independent experiments ± SD. (b) MTT colorimetric assay (over a time period of 60 h) comparing MAPT overexpressing Rin-5F cell line versus Empty; *p* < 0.05. Presented data are means calculated from three independent experiments ± SD.

Already when establishing both cell lines it came into our attention that a higher growth rate might be specific to the transgene expressing cell line. This observation could be ascertained by assessing proliferation rate by cell counting. MAPT overexpression in this cell line significantly accelerates (*p* < 0.05) cell growth by about 25% (Figure 5(a)).

In addition, the colorimetric MTT assay was determined which demonstrated about 60% higher activity rate of the MAPT overexpressing cell line when compared with Empty cell line (*p* < 0.05) (Figure 5(b)).

### 3.7. Influence of MAPT Overexpression on Gene Expression

**Figure 6:**
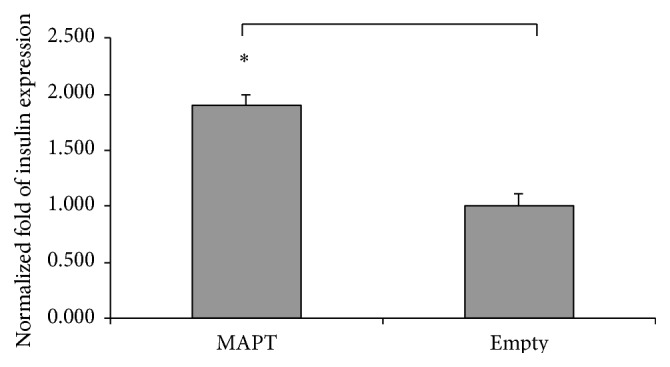
RT-qPCR of RNA isolated from Rin-5F.* Ins1* gene expression in MAPT overexpressing (*n* = 4) versus Empty cell line (*n* = 4) was evaluated using the ΔΔCT method; *p* < 0.05.

It was previously shown that MAPT binds to DNA and thus it most probably affects chromatin [41, 42]. To evaluate the influence of MAPT overexpression on insulin gene expression, we performed RT-qPCR with both cell lines and observed about twofold higher levels of* Ins1* gene transcription in the MAPT overexpressing cell line (*n* = 4) (SD ± 0.15) compared to Empty (*n* = 4) (SD ± 0.09) (*p* < 0.05) (Figure 6).

### 3.8. Influence on Insulin Secretion Processes

MAPT has an influence on microtubule stabilization. The tubular transport of secretory granules is a key player in endocrine secretory processes. Therefore, measurement of insulin secretion of the transduced cell lines was examined.

At first, we determined the total insulin content, and the results confirmed the RNA data: the total insulin content was much higher in MAPT overexpressing cells (52.6 ± SD 4.18 ng/2 × 10^5^ cells) than in Empty (24.5 ± SD 2.68 ng/2 × 10^5^ cells), *p* < 0.05.

**Figure 7:**
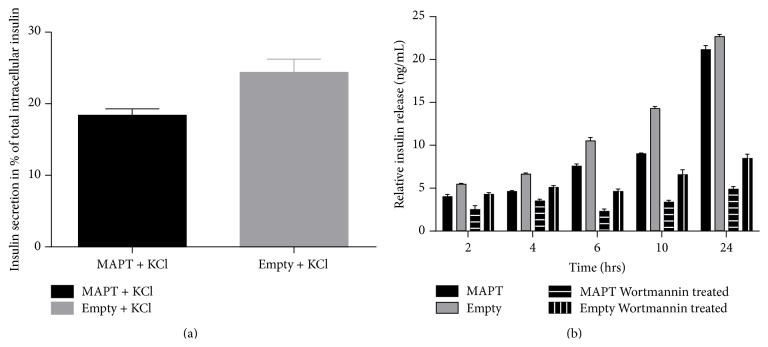
Insulin secretion. (a) Insulin secretion after stimulation for 2 h with 20 mM KCl. Insulin secretion from MAPT overexpressing cells versus Empty was calculated in the percentage of total intracellular insulin content; *p* < 0.05. (b) MAPT overexpressing Rin-5F cell line versus Empty over a time period of 24 hours. Cell lines were either grown in RMPI 1640 medium or in RMPI 1640 medium supplemented with 1 *μ*M Wortmannin. Each column representing relative insulin secretion level is obtained for respective cell line as described in the legend attached directly to the graph. The insulin secretion was normalized to the total intracellular insulin content. Data is presented from 3 independent experiments ± SD.

Interestingly, when the insulin release from MAPT overexpressing versus Empty cell lines was measured upon stimulation with 20 mM KCl, the MAPT overexpressing cells showed significantly lower insulin secretion rates (Figure 7(a)) when calculated in the percentage of the total intracellular insulin content. This lower percentage of insulin release in MAPT overexpressing cells was not only observed under stimulated conditions, but it was also observed under normal culture conditions.

Graph represents a mean of three independent experiments ±SD.

### 3.9. Wortmannin Treatment

In order to assess whether MAPT phosphorylation dynamics is important for anterograde transport and thereby for insulin trafficking, we included the phosphatidylinositol 3-kinase (PI3K) inhibitor, Wortmannin [43]. Inhibition of PI3K leads to the overactivation of glycogen synthase kinase 3*β* (GSK-3*β*) and consequently to MAPT hyperphosphorylation. Application of Wortmannin causes strong MAPT hyperphosphorylation in both Rin-5F cell lines (data not shown).

Interestingly, one *μ*M Wortmannin reduced significantly the insulin secretion rates in both treated cell lines, *p* < 0.05 (Figure 7(b)). The insulin secretion rates were evaluated in ± Wortmannin treated MAPT overexpressing and Empty cell lines during the period of 24 h. This data could reflect the importance of balanced MAPT phosphorylation-dephosphorylation status. Graph represents the mean of three independent experiments ±SD.

### 3.10. MAPT Knockdown

**Figure 8:**
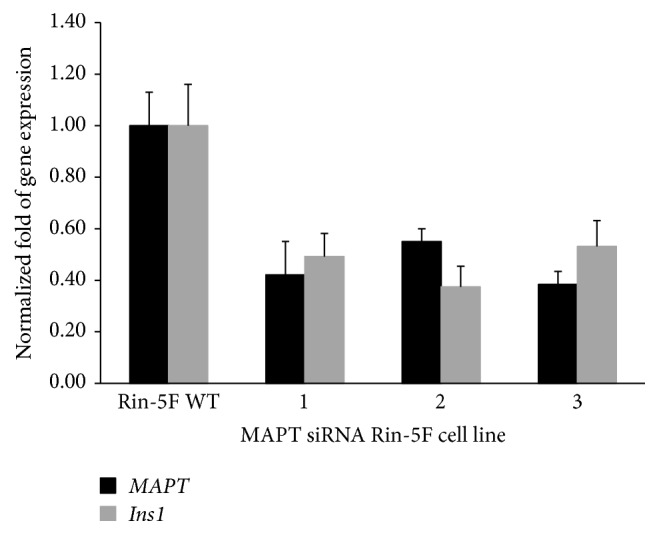
MAPT knockdown. RT-qPCR of RNA isolated from three different MAPT siRNA transduced Rin-5F cell lines (marked as numbers 1, 2, and 3). The rat endogenous* Mapt* and* Ins1* gene expression level was evaluated using the ΔΔCT method.

To further investigate the role of MAPT in insulin trafficking, we aimed to knock down endogenous* Mapt* gene in the Rin-5F insulinoma cell line. For that reason, we transduced 4 different* Mapt* small interfering RNAs (siRNAs) encoding plasmids separately by means of lentiviral approach into the Rin-5F insulinoma cell line. However, these transduced cell lines had a slow division rate and higher apoptotic rate than wild type cells. Nevertheless, we evaluated the silencing efficiency at the mRNA level by RT-qPCR (Figure 8) reaching up to 60% of* Mapt* knockdown. Furthermore, we examined if partly silenced* Mapt* influences insulin expression (Figure 8): in three out of four cell lines, the insulin gene expression level was significantly reduced (up to 60%) going in line with the* Mapt* gene knock-down effects.

Consequently, we assessed protein expression level by Western blotting. This confirmed reduction of MAPT protein expression (data not shown).

## 4. Discussion

In the present study, we examined MAPT, which represents one of the most important molecules for microtubule dynamics and trafficking within the cell. We demonstrated MAPT expression in human pancreatic islets of Langerhans and its upregulation in human insulinomas. Moreover, we studied its relevance in pancreatic islets using the beta-cell derived rodent Rin-5F cell line transduced with one of the MAPT isoforms (3-repeat domain MAPT, namely, MAPT3). Our data suggest an influence of MAPT on insulin transcription, secretion, and growth dynamics of the Rin-5F cell line.

MAPT plays an important role in microtubule stabilization and neurite outgrowth in the CNS [8, 36, 37, 44]. If MAPT is abnormally hyperphosphorylated it cannot exert its function, and this loss of function ultimately leads to neuronal pathology including disintegration of axonal tubulin [45–47]. Neuropathologically, these changes are present as intracytoplasmic NFTs and as NTs in axons and dendrites; NFTs and NTs are hallmark lesions of AD and may also be seen in other tauopathies [48–50]. In line with the notion of MAPT expression in non-CNS tissue including the pancreas [16, 38], an analogy between AD and type 2 diabetes mellitus (T2DM) has been suggested [51–54], in particular with respect to insulin signaling pathway dysfunction, protein aggregation, and oxidative events. We have previously demonstrated MAPT expression in a pancreatic beta-cell line model, human pancreatic islets of Langerhans, and human insulinomas [20] and described biochemical features of pancreatic MAPT that are somewhat similar to the hyperphosphorylated MAPT in AD brains.

In the present study, we further characterized pancreatic MAPT to better understand the mechanisms leading to dysregulation in pancreatic endocrine cells. At first, we evaluated the MAPT expression profile in the human islets of Langerhans from healthy organ donors and from human insulinomas. We found that MAPT3 (381 amino acids) and MAPT4 (412 amino acids) isoforms occur at much higher frequency than other MAPT splice variants which we have demonstrated at the mRNA and the protein level. Our finding of 3 times higher MAPT expression in pancreatic insulinomas compared to healthy islets suggests that MAPT might be involved in insulinoma tumor biology.

It has been shown that, in respect to the microtubule binding domain, MAPT occurs in two splice variants containing either 3- (3RD) or 4- (4RD) repeat domain. Tauopathies in the CNS are characterized by the presence of either 3RD MAPT (e.g., Pick's disease) or 4RD MAPT (e.g., progressive supranuclear palsy) or both in imbalanced ratio (e.g., AD) [54–59]. Strikingly, we found an imbalance in human insulinomas (ratio 3RD : 4RD >1), whereas no disproportion was found in human islets. These findings suggest that the imbalance between 3RD and 4RD MAPT isoforms might be of clinical relevance in insulinomas.

In order to find further proof for this hypothesis, we studied MAPT in a beta-cell derived rodent insulinoma Rin-5F cell line, which represents a model for the evaluation of mechanisms controlling transcription, storage, and secretion of insulin.

Therefore, we cloned one of the MAPT isoforms; we have chosen the shorter isoform 3RD MAPT (381aa, named also MAPT3) as this occurred in human insulinomas in higher copy number than 4RD. After successful generation of a stable MAPT overexpressing Rin-5F cell line, we studied the potential influence of MAPT on cell growth and on insulin transcription and secretion.

We found higher cell replicative potential at the MAPT overexpressing cell line which was reflected in much higher MTT activity. Interestingly, the proliferation data could reflect the human clinical data: high MAPT expression level in human insulinomas is going in line with an elevated growth rate of tumor tissue. Noteworthy, the Rin-5F cell lines transduced with plasmids encoding MAPT siRNAs had higher apoptotic rates than wild type Rin-5F and also much slower growth rate. Our findings at the* in vitro* pancreatic beta-cell models support the suggestion that MAPT has some influence on the biology of these cell lines, especially on theirs growth dynamics.

Furthermore, as it was previously demonstrated* in vitro* and* in vivo* that MAPT interacts with DNA [42], we hypothesized that MAPT overexpression could cause alterations at nuclear level in Rin-5F cells. Indeed, the RNA analysis confirms that the MAPT overexpressing cell line has about 2 times higher expression of the insulin gene. Moreover, the insulin protein level confirmed the mRNA data as the MAPT overexpressing cell line displayed about twice as high insulin content when compared to Empty.

Since MAPT is a major player in the microtubule transport machinery, and as the tubular transport of secretory granules is fundamental for endocrine secretion, we evaluated insulin release. According to our data, the relative amount of secreted insulin from the MAPT overexpressing cell line is lower when taken as a percentage of total intracellular insulin. Our finding is in line with earlier studies on nonneuronal cell types overexpressing MAPT where they have shown that this protein inhibits anterograde organelle transport [60, 61]. Ebneth suggested that MAPT by binding to microtubules slows down intracellular transport [60].

Interestingly, the upregulation of glycogen synthase kinase 3*β* (GSK3*β*), a main kinase influencing MAPT phosphorylation status, results in MAPT hyperphosphorylation and, subsequently, the distortion of the anterograde transport [62].

In this respect, the continuous phosphorylation and dephosphorylation of MAPT protein are crucial for its biological function. Hence, we intended to influence the continuous phosphorylation and dephosphorylation of MAPT protein by applying Wortmannin. Interestingly, we saw a marked reduction in insulin secretion under time dependent Wortmannin application. We suspect that hyperphosphorylated MAPT cannot bind to microtubules and stabilize them; therefore, insulin granules trafficking is disturbed. Our data support the hypothesis that a balance between the levels of GSK3*β* and MAPT phosphorylation is required to maintain anterograde transport.

In further studies on this subject it might be of interest to investigate the posttranslational modifications, especially phosphorylation and glycosylation of MAPT in various forms of diseases in which insulin secretion is changed. This concerns neuroendocrine cells by T2DM, insulinoma, and Alzheimer's disease.

To gain further insight into the importance of MAPT in insulin trafficking, we attempted to knock down MAPT by means of siRNAi. Successful knocking down MAPT at the gene level was followed by marked reduction in insulin gene transcription. Of note, we encountered problems with growing the MAPT siRNA transduced cell lines and did not perform insulin secretion assays, as the cell number was limited due to high apoptosis rates. We think that a secretion assay under such unfavorable conditions is unreliable.

Taken together, our data strongly indicate the importance of MAPT for the pancreatic beta-cell. On the one hand, MAPT might have an influence on a pancreatic tumor formation and its outgrowth. Not only the elevated level of MAPT transcription rates in tumor tissue, but also a disproportion between 3RD and 4RD MAPT isoforms might have an impact on pathological changes. On the other hand, MAPT might influence the anterograde transport and thus the insulin trafficking from the beta cells.

However, further studies are warranted to identify in more detail the role of MAPT in insulin exocytosis.

## 5. Conclusions

In this study, we demonstrate significant upregulation of MAPT gene and protein expression in human insulinoma tumor tissues when compared to healthy islets of Langerhans. Our data support the hypothesis that MAPT may be one of the key players contributing to growth dynamics of beta-cell derived rodent Rin-5F cell line. Furthermore, we find that MAPT influences insulin gene transcription rate and might be an important protein in insulin trafficking. We conclude that the disturbed phosphorylation and dephosphorylation status of MAPT contribute to the deregulation of insulin secretion.

## Abbreviations

MAPT: Microtubule-associated protein tau
AD: Alzheimer's disease
GSK-3*β*: Glycogen synthase kinase 3*β*
RD: Repeat domain
T2DM: Type 2 diabetes mellitus
CNS: Central nervous system.
